# Investigation of the mechanisms and experimental verification of *Cuscuta-Salvia* in the treatment of polycystic ovary syndrome (PCOS) via network pharmacology

**DOI:** 10.1186/s13048-022-00964-8

**Published:** 2022-04-04

**Authors:** Ying-ying Zhang, Jian-xiong Ma, Yu-tian Zhu, Yi-xuan Wang, Wang-qiang Chen, Xin Sun, Wei Zhang, Chen-ye Wang, Cai-fei Ding

**Affiliations:** 1 The Second Clinical Medical College, Zhejiang Chinese Medical University, Hangzhou, China; 2 Department of Traditional Chinese Medicine, Peking University Third Hospital, Beijing, China; 3 Department of Reproductive Medicine, Zhejiang Provincial Integrated Chinese and Western Medicine Hospital, Hangzhou, China

**Keywords:** *Cuscuta-Salvia*, PCOS, Network pharmacology, Ovary, Core gene

## Abstract

Polycystic ovary syndrome (PCOS) is an endocrine disease associated with reproduction. The *Cuscuta-Salvia* formula has been widely used to treat for PCOS in clinic. However, its chemical and pharmacological properties remain unclear. We identified the active components and related targets of *Cuscuta-Salvia* using UHPLC-ESI-Q-TOF-MS and TCMSP database. Disease targets were obtained from the DisGeNET and GeneCards databases. Subsequently, common targets between *Cuscuta-Salvia* and PCOS were identified using a Venn diagram. PPI network was established. Core genes were selected using a Cytoscape software plugin. GO and KEGG enrichment analyses were performed for common targets using the “pathview” package in *R*. Several core targets were verified using molecular and Immunological methods. By combining UHPLC-ESI-Q-TOF-MS with a network pharmacology study, 14 active components and a total of 80 common targets were obtained. Ten core genes were regulated by *Cuscuta-Salvia* in PCOS, including IL6, AKT1, VEGFA, TP53, TNF, MAPK1, JUN, EGF, CASP3, and EGFR. GO results showed that cellular response to drugs, response to oxygen levels, response lipopolysaccharides, and response to molecule of bacterial origin in BP category; membrane, transcription regulator complex, nuclear chromatin, postsynaptic membrane, and vesicle lumen in CC category; DNA-binding transcription factor binding, RNA polymerase II-specific DNA-binding transcription factor binding, DNA-binding transcription activator activity, RNA polymerase II-specific, DNA-binding transcription activator activity, and cytokine receptor binding in MF terms. The KEGG enrichment pathway was mainly involved in the PI3K − Akt, MAPK, TNF, IL-17 signalling pathways, and in cellular senescence. Furthermore, the results of the experimental study showed that *Cuscuta-Salvia* ameliorated the pathological changes in the ovaries, liver and adipose tissue. And it improved the expressions of the genes or proteins. Our results demonstrate that *Cuscuta-Salvia* may provide a novel pharmacological basis in an experimental model of PCOS by regulating gene expression. This study provides a basis for future research and clinical applications.

**Supplementary Information:**

The online version contains supplementary material available at 10.1186/s13048-022-00964-8.

## Introduction

Polycystic ovary syndrome (PCOS) is a common and complex endocrine disorder, with patients suffering from androgen excess, oligomenorrhoea or amenorrhoea, and polycystic ovaries. These conditions are related to insulin resistance, metabolic abnormalities and infertility [9]. It has been reported that 18% of pharmacological women are affected by PCOS [20]. Currently, PCOS treatment mainly consists of anti-androgen drugs, insulin sensitisers, and ovulation-promoting drugs [54].The aetiology of PCOS remains unclear and its process is very complex [28]. However, traditional Chinese medicine (TCM) has multiple targets for the treatment of some diseases.

TCM has been widely applied in the treatment of PCOS in China [30], in which *Cuscuta-Salvia* (*Semen Cuscutae*-*Radix Salviae*), a common Chinese herbal formula is used to treat PCOS. *Cuscuta* (Semen Cuscutae; Tusizi in Chinese) belongs to the Convolvulaceae family and is a holoparasitic angiosperms [19]. Accumulating evidence has shown that *C. chinensis* flavonoids, the main components of *Cuscuta*, effect endocrine function, including the improvement of reproductive hormone levels [15], oestradiol [47], and glycolipid metabolism [33]. According to TCM theory [26], *Cuscuta* nourishes the liver and kidney, and benefits Yin. The other herb is *Salvia* (Radix Salviae; Danshen in Chinese). *Salvia* belongs to the Lamiaceae family [32], and tanshinone is extracted from its roots*.* Previous studies have reported that tanshinone can improve body weight, incompact ovarian follicles, and the levels of reproductive hormones (testosterone, androstenedione, luteinising hormone, etc.) [53]. According to TCM theory [13], *Salvia* relieves menstruation symptoms and pain. Thus, based on these properties, we speculated that the combination of *Cuscuta* and *Salvia* could improve PCOS.

Recently, network pharmacology has been used to uncover the active components and potential mechanisms of Chinese herb pairs according to the theory of systems biology. In this study, we hypothesised that the multi-target mechanisms of *Cuscuta-Salvia* against PCOS could be demonstrated using network pharmacology and verified these mechanisms in experiments. The study aimed to screen out these bioactive components and targets of PCOS using network pharmacology. Subsequently, animal experimental verification was carried out to investigate the relationship between candidate genes and potential mechanisms of PCOS, which will enhance the possibility of using *Cuscuta* and *Salvia* in the treatment of PCOS.

## Materials and methods

### Analysis of *Cuscuta-Salvia* using UHPLC-ESI-Q-TOF-MS

Ultra-high-performance liquid chromatography coupled with electrospray ionization quadrupole time-of-flight mass spectrometry (UHPLC-ESI-Q-TOF-MS) analysis was conducted to identify the active components of *Cuscuta-Salvia*. The conditions were as follows: the chromatographic analysis of *Cuscuta-Salvia* was performed on an ACQUITY UPLC BEH C_18_ column (2.1 × 100 mm, 1.7 μm; Waters Corporation, Milford, MA, USA) at a flow rate of 0.3 mL/min and room temperature. The volume of each injection was 3 μL. The mobile phase was composed of acetonitrile (A) and 0.1% formic acid (B): 0–2 min, 95% B; 2–22 min, 95–0% B; 22–23 min, 0–0% B; 23–23.5 min, 0–95% B; 23.5–25 min, 95–95% B. A TurboIon Spray ion source and electrospray ionization positive and negative ion scanning mode were used to perform the time of flight mass spectrometry (TOF-MS). The optimal TOF-MS conditions were as follows: sample cone was 40 kV; source offset 80 kV; source temperature, 100 °C; desolvation temperature, 400 °C; cone gas, 50 L/h; desolvation gas, 800 L/h; nebuliser, 6.0 bar; scanning time, 0.2 s/spectra; and scanning m/z range, 50–1200 Da. Under the positive and negative ion modes, the capillary voltage was 3.0 kV and 2.5 kV, respectively. In this study, the active components were identified using SCIEX OS software 1.4, based on the first-order accurate mass number, isotope distribution ratio, and MS/MS of the components.

### Identification of the active components of *Cuscuta-Salvia*

To collect the active components of *Cuscuta-Salvia*, we used databases, including the Traditional Chinese Medicine Systems Pharmacology (TCMSP, https://tcmspw.com/tcmsp.php, version 2.3) [40].

Based on the properties of absorption, distribution, metabolism, and excretion (ADME), we screened and identified bioactive components, removing any pharmacological compounds with poor pharmacological properties [50]. Thus, to obtain the fully active components, we adopted two conditions as the criteria to screen the candidate components, including oral bioavailability (OB) ≥ 30% and drug-likeness (DL) ≥ 0.18 [27]. The results were compared with the results of the UHPLC-ESI-TOF-MS analysis. Finally, the component prediction targets of the two databases were combined to establish a database of the chemical constituents of *Cuscuta-Salvia*.

### Collection of PCOS candidate genes

We used “polycystic ovary syndrome” as index keywords, and the selected species were limited to “*Homo sapiens*” in search of therapeutic targets for PCOS. To obtain candidate genes for PCOS, databases of DisGeNET (http://www.disgenet.org/) and GeneCards (http://genealacart.genecards.org/) (relevance scores of 5 or more) were performed [45]. Duplicates of candidate gene results were removed to obtain a target related to PCOS.

### Establishment of an herb-compound target-PCOS target network of *Cuscuta-Salvia*

Common targets between herbs and diseases were generated by establishing a Venn diagram (https://bioinfogp.cnb.csic.es/tools/venny/) of their intersecting gene symbols. Then we used Cytoscape v3.7.2 (www.cytoscape.org/) to construct the herb-compound target-PCOS target network of *Cuscuta-Salvia* [51]. In the network, herbs, bioactive compounds, and their related targets were expressed as nodes, while the interactions between nodes were expressed as edges. The “degree” value of the node is the number of links connected to the node. The larger the degree value is, the more important the target is.

### Establishment of a protein-protein interaction (PPI) network

To interpret the interactions between target proteins, the common targets were input into the STRING (http://string-db.org) online website to obtain a protein-protein (PPI) network. Protein interactions with a confidence score > 0.4 were analysed [45]. Next, the nodes and score information were input into the Cytoscape v3.7.2 software for visual analysis. According to Cytohubba plugin, the top 10 genes were obtained. Potential targets were then predicted.

### Gene Ontology (GO) functional enrichment analysis and Kyoto Encyclopedia of Genes and Genomes (KEGG) pathway enrichment analysis

Using the “pathview” package in *R* for *Cuscuta-Salvia* in the treatment of PCOS, GO functional analysis and KEGG pathway enrichment analysis were performed for common targets. GO functional analysis is mainly used to annotate gene functions, including three ontologies: biological processes (BP), cellular components (CC), and molecular functions (MF) [10, 11]. KEGG enrichment analysis was useful for the enrichment analysis of common PCOS targets [21]. *P* < 0.05 was recognised as statistically significant for both the GO and KEGG analyses.

### Preparing for animal models of PCOS in mice

Twenty-four female C57BL/6 mice (~ 3 week old) were obtained from the Animal Centre of Zhejiang Chinese Medical University (Hangzhou, Zhejiang, China), Certificate NO: SYXK (Zhejiang Province) 2021–0012. Mice were housed in a Specific Pathogen Free (SPF)-degree facility (20–26 °C and 12-h light/dark cycle). Standard fodder and tap water were available in the mouse cages at the Laboratory Animal Research Centre for 1 week prior to the experiment. All the procedures were approved by the Animal Ethics Committee, Zhejiang Chinese Medical University.

All mice (~ 4 week old) were randomly divided into 4 groups: normal control group (NC), model group (Model), *Cuscuta-Salvia* group (CS), and metformin group (Met), with 6 mice in each group. After adaptive feeding for 1 week, all mice (except for NC) were intragastrically (i.g.) administrated of letrozole solution (3 mg/kg) [56] and NC mice were i.g. administrated of normal saline for 1 month, respectively. Meanwhile, CS mice were oral given 0.1 mL/10 g of *Cuscuta-Salvia* extraction, Met mice were oral given 50 mg/kg of metformin, and the other mice were oral given the same amount of normal saline for 30 days. Signs of successful modelling included disordered oestrous cycle stages, as evidenced by vaginal smears (Supplementary Fig. 1). On day 26, After the modelling and treatment were completed, all mice were euthanised by intraperitoneal injection of sodium pentobarbital and subjected to remove mice ovaries and other tissues to perform the further experiments.

### PCOS mouse weight

The body weight of the mice was measured every day, ranging from day 1 to day 25.

### Oral glucose tolerance test (OGTT) assay [36]

After being fasted for 12 h, the blood glucose of all mice was measured using an ACCU-CHEK Performa at 9:00 a.m. All mice were administered a glucose solution (2 g/kg body weight) orally. One drop of fresh blood was collected from the tail of each mice using a glucometer to measure the glucose concentration. Meanwhile, measurements were performed at 0, 30, 60, 90, and 120 min [52]. The total area under the glucose response curve (AUC_0–2h_) was calculated using GraphPad Prism 6 software.

### Haematoxylin and eosin (HE) staining [8]

The ovary tissues, liver tissues, and adipose tissues obtained from the mice were fixed in 4% PBS-paraformaldehyde solution (pH 7.4) at room temperature for 48 h and embedded in paraffin. After dehydration and clearing, the tissues were immersed in wax and cut into 5-μm-thick slices. The slices were dewaxed and stained with HE. Six mice in each group were used for the HE experiments.

### Determination of quantitative real-time PCR (qRT-PCR) [31]

Total RNA was extracted from the ovary tissues of mice using TRIzol reagent (Ambion RNA; Life Technologies). cDNA was synthesised using a FastKing RT Kit (with gDNase). Gene expression was determined by quantitative real-time PCR (qRT-PCR) using SYBR Green Master Mix (Bio-Rad), and the primer sequences are listed in Table 1. The qRT-PCR procedure was performed as follows: 40 cycles of UDG activation at 50 °C for 2 min, Dual-Lock™ DNA polymerase at 95 °C for 2 min, denaturation at 95 °C for 15 s, annealing at 58 °C for 15 s, and elongation at 72 °C for 1 min. Relative quantification was performed using the comparative Ct (2^-ΔΔCt^) method method [39]. Three mice in each group are used for qRT-PCR experiments.

**Table 1:** The primers for qRT-PCR

ID	Sequence (5′-3′)	Product Length (bp)
β-actin β-actin	F: TGTGATGGTGGGAATGGGTCAGAA R: TGTGGTGCCAGATCTTCTCCATGT	140
IL6 IL6	F: CCAAGAGGTGAGTGCTTCCC R: CTGTTGTTCAGACTCTCTCCCT	118
Akt1 Akt1	F: ATGAACGACGTAGCCATTGTG R: TTGTAGCCAATAAAGGTGCCAT	116
TP53 TP53	F: GCGTAAACGCTTCGAGATGTT R: TTTTTATGGCGGGAAGTAGACTG	144
MAPK1 MAPK1	F: CCCAAGTGATGAGCCCATTG R: TCAATGGAAGGGGACAAACTGA	248
JUN JUN	F: TCGCTCGGCTAGAGGAAAAA R: CTGCTGCGTTAGCATGAGTT	152
EGF EGF	F: AGGAGGTCCGCTAGAGAAATG R: CAGGCGATGAACAACCAGTG	279
CYP17a1 CYP17a1	F: CTCCAGCCTGACAGACATTC R: CTGAGAACACACTTGGGTCC	89
CYP19a1 CYP19a1	F: GGGGACAGTATGCTGGCTAA R: GCCCAGCTTCTCCCTGTAAA	282
AR AR	F: CGAAGTGTGGTATCCTGGTG R: CTGGTACTGTCCAAACGCAT	132
FSHb FSHb	F: CTGGTGCTGGAGAGCAATCT R: ACTTTCTGGGTATTGGGCCG	174
LHb LHb	F: AACTCCCAAGCATCAGCCTC R: AGATGTGGAGGTGGCTAGAG	171

### Western blot analysis

Proteins were extracted from the ovary tissues of mice using RIPA buffer containing protease inhibitors. Total protein was separated by 10% sodium dodecyl sulphate-polyacrylamide gel electrophoresis and transferred to polyvinylidene fluoride membranes. The membranes were then incubated with the following specific primary antibodies obtained from Cell Signaling Technology (Danvers, MA, USA): TP53 (rabbit, 1:1000), p-AKT (rabbit, 1:1000), AKT (rabbit, 1:1000), p-MAPK (rabbit, 1:1000), and MAPK (rabbit, 1:1000). The membranes were then incubated with HRP-conjugated secondary antibodies. Eventually, the protein bands were photographed and images were developed.

### Immunohistochemistry (IHC) staining

**Fig. 1:**
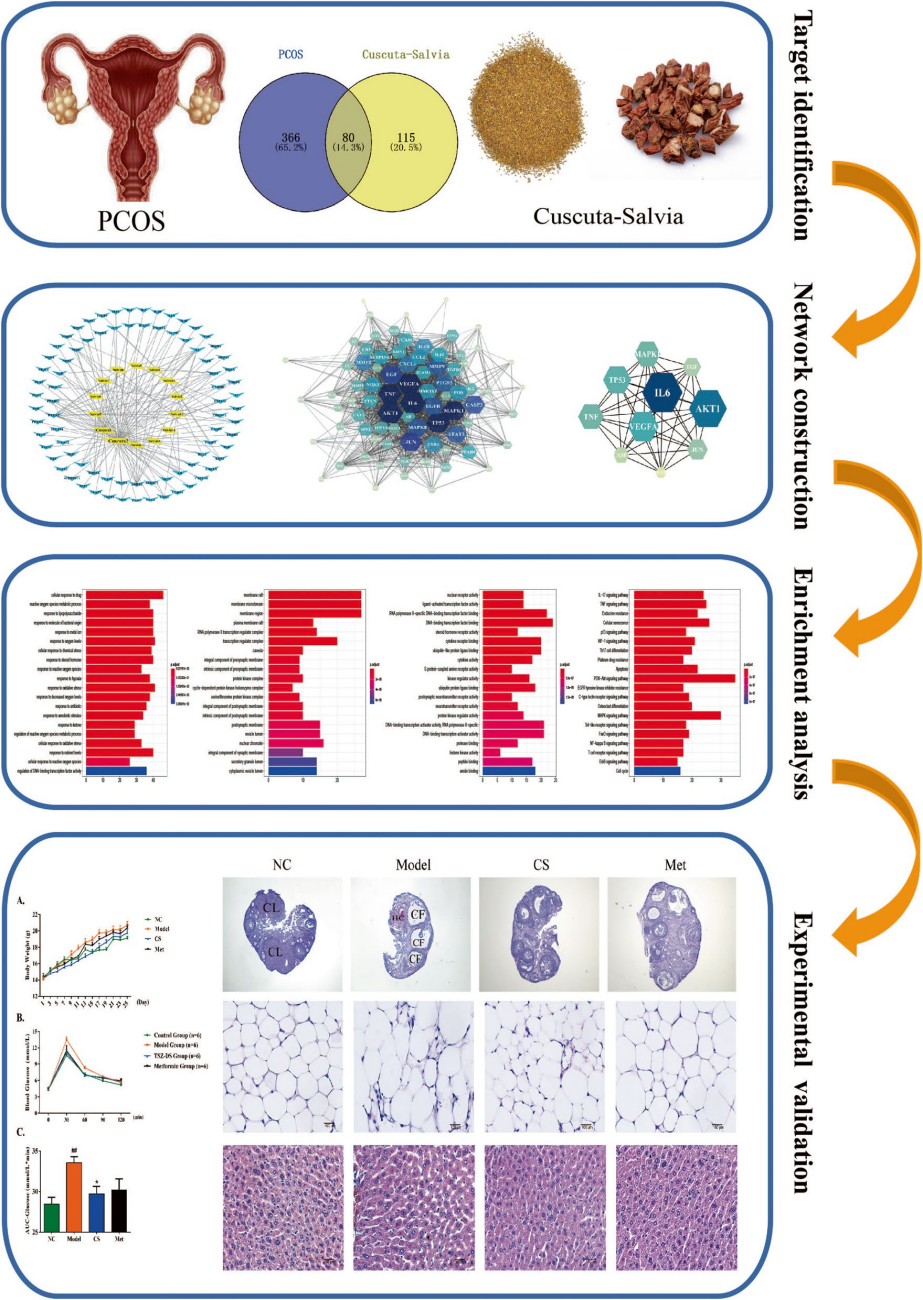
Flow diagram of the network pharmacological research upon the *Cuscuta-Salvia* against PCOS

The sections were then incubated with each primary antibody. Primary antibodies against c-JUN (rabbit, 1:200; Cell Signaling Technology) and VEGFA (rabbit, 1:200; Cell Signaling Technology) were used in this step. Next, the slices were incubated with secondary antibodies (HRP-conjugated goat anti-rabbit immunoglobulins) for 0.5 h at 37 °C, and finally observed and photographed using a fluorescence microscope. The workflow is illustrated in Fig. 1.

### Statistical analysis

All data are shown as the mean ± standard error of the mean ($\overline{x}$ ±SEM). Statistical data were processed using IBM SPSS Statistics for Windows, version 25.0 (IBM, Armonk, NY, USA) and GraphPad Prism 6. One-way analysis of variance was used to determine the least significant difference in each group (homogeneity of variance and conformation to normal distribution). Otherwise, Dunnett’s test was used to determine the significant differences between groups. Differences were considered statistically significant at *P* < 0.05.

## Results

### Identification for the active components of *Cuscuta-Salvia*

**Fig. 2:**
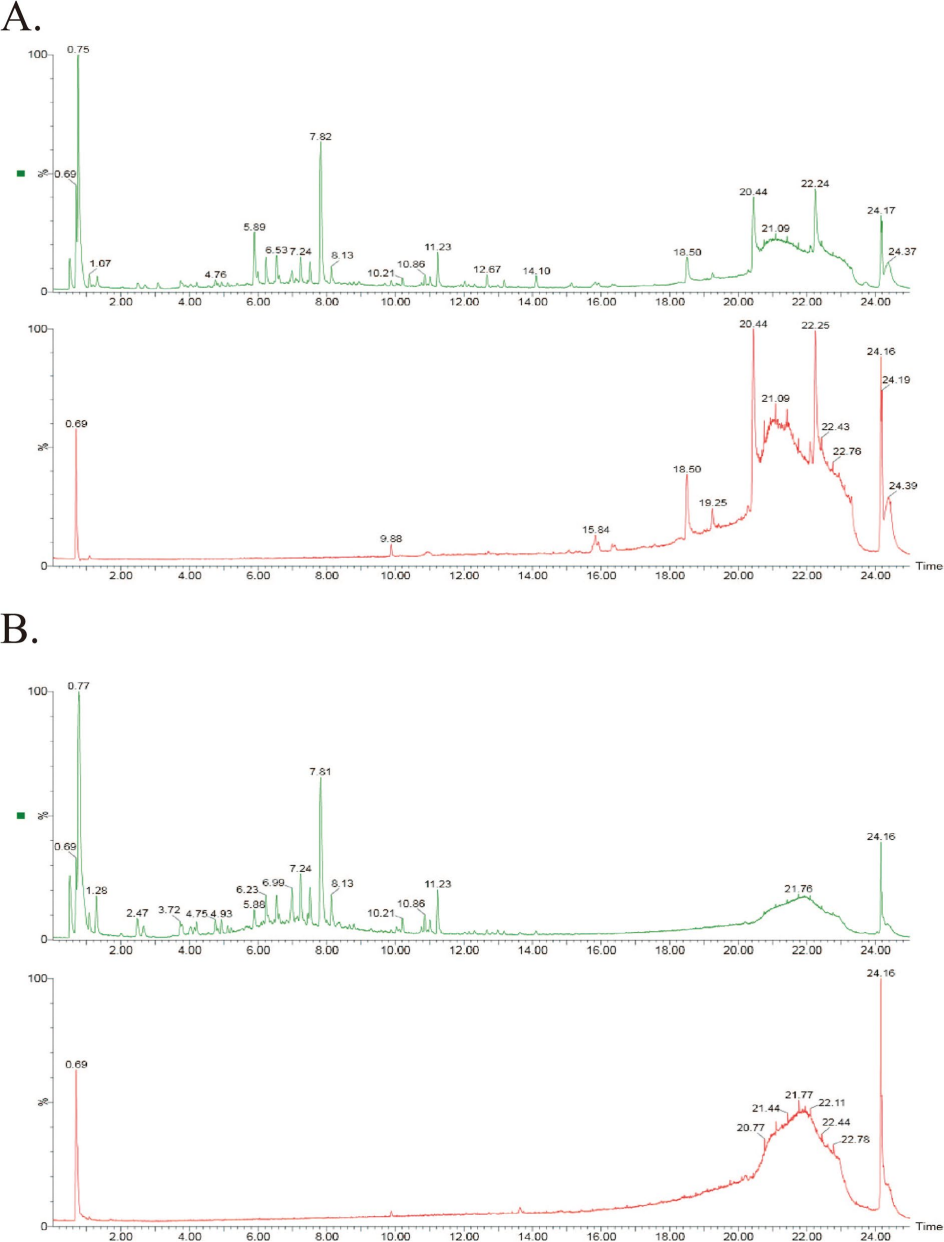
The total ion flow diagram of *Cuscuta-Salvia* by UHPLC-ESI-Q-TOF-MS. **A** Positive ion mode; **B** negative ion mode

The total ion flow diagram of *Cuscuta-Salvia* was obtained from the UHPLC-ESI-Q-TOF-MS analysis, and the compounds were identified qualitatively by using SCIEXOS software 1.4. As shown in Fig. 2 and Table 2, 12 compounds were identified under in positive ion mode and 14 compounds were identified under in negative ion mode.

**Table 2:** High resolution mass spectrometry data and elemental composition of *Cuscuta-Salvia.* (No. 1–14 was under negative ion mode and 15–26 was under positive ion mode)

No.	Molecular Name	Molecular Formula	Retention Time (min)	m/z	Measure Molecular Mass (Da)	Molecular Mass (Da)
1	Methyl tanshinate	C_20_H_18_O_5_	1.29	383.1137	338.1155	338.11542
2	Caffeate	C_9_H_8_O_4_	2.48	179.0354	180.0427	180.04226
3	Neocuscuscutosside A	C_37_H_46_O_21_	4.75	871.2478	826.2496	826.25316
4	Cuscutoside A	C_31_H_36_O_16_	5.89	663.1916	664.1989	664.20034
5	Salvianolic acid A	C_26_H_22_O_10_	6.2	539.12	494.1218	494.1213
6	Salvianolic acid D	C_20_H_18_O_10_	6.54	463.0889	418.0907	418.09
7	Miltionone II	C_19_H_20_O_4_	6.96	357.134	312.1358	312.13616
8	Danshensu	C_9_H_10_O_5_	7.24	197.046	198.0533	198.05282
9	Isoimperatorin	C_16_H_14_O_4_	7.51	269.085	270.0923	270.08921
10	Tanshinone IIA	C_19_H_18_O_3_	7.87	339.1273	294.1291	294.12559
11	Isotanshinone I	C_18_H_12_O_3_	8.13	311.0488	276.0794	276.07864
12	dan-shexinkum d	C_20_H_18_O_4_	10.2	371.108	336.1386	336.13616
13	Danshinspiroketallactone	C_18_H_22_O_3_	11.21	331.1562	286.158	286.15689
14	Methylenedihydrotanshin-quinone	C_18_H_16_O_3_	11.22	325.1086	280.1104	280.10994
15	Protocatechualdehyde	C_7_H_6_O_3_	2.5	139.0393	138.0321	138.03169
16	Hydroxytanshinone IIA	C_19_H_18_O_4_	5.89	333.1086	310.1194	310.12051
17	Quercetin-3-O-β-D-gluco-pyranoside	C_21_H_20_O_12_	6.23	465.1032	464.0959	464.09548
18	Quercetin	C_15_H_10_O_7_	6.54	303.0502	302.0429	302.04265
19	Kaempferol	C_15_H_10_O_6_	7.09	287.0555	286.0482	286.04774
20	Lithospermic acid B	C_36_H_30_O_16_	7.24	736.1883	718.1545	718.15338
21	Salvianolic acid G	C_26_H_22_O_10_	7.52	341.0662	340.0589	340.0583
22	Prolithospermic acid	C_27_H_22_O_12_	7.83	539.1203	538.113	538.11113
23	15, 16-dihydrotanshinone I	C_18_H_14_O_3_	12.67	279.1009	278.0936	278.09429
24	Tanshinone IIB	C_19_H_18_O_4_	14.11	297.1488	296.1415	296.14124
25	Cryptotanshinone	C_19_H_20_O_3_	15.13	297.1491	296.1418	296.14124
26	Isocryptotanshi-none	C_19_H_20_O_3_	13.18	297.1492	296.1419	296.14124

### Screening of the active components of *Cuscuta-Salvia*

A total of 231 chemical compounds of between *Cuscuta* and *Salvia* were collected from the traditional Chinese medicine systems pharmacology database and analysis platform (TCMSP), in which included 30 compounds in *Cuscuta* and 202 compounds in *Salvia.* Combined with the parameters of OB ≥ 30% and DL ≥ 0.18 in TCMSP, 69 potential active components were screened by removing duplicate values, including 10 compounds related to *Cuscuta* and 59 compounds related to *Salvia*. In addition, the filtered results in TCMSP and the results of UHPLC-ESI-Q-TOF-MS intersected. Thus, 14 active components were identified in this study, which are listed in Table 3.

**Table 3:** *Cuscuta* and *Salvia* active components list

Herb	MOL ID	Molecule Name No.	Molecular Formula
*Cuscuta*	MOL000422	19	C_15_H_10_O_6_
*Cuscuta*	MOL000098	18	C_15_H_10_O_7_
*Salvia*	MOL001942	9	C_16_H_14_O_4_
*Salvia*	MOL007101	23	C_18_H_14_O_3_
*Salvia*	MOL007154	10	C_19_H_18_O_3_
*Salvia*	MOL007045	16	C_19_H_18_O_4_
*Salvia*	MOL007155	24	C_19_H_18_O_4_
*Salvia*	MOL007088	25	C_19_H_20_O_3_
*Salvia*	MOL007108	26	C_19_H_20_O_3_
*Salvia*	MOL007120	7	C_19_H_20_O_4_
*Salvia*	MOL007093	12	C_20_H_18_O_4_
*Salvia*	MOL007141	21	C_26_H_22_O_10_
*Salvia*	MOL007130	22	C_27_H_22_O_12_
*Salvia*	MOL007111	11	C_18_H_12_O_3_

### Screening of the candidate genes of the active compounds in *Cuscuta-Salvia*

A total of 404 candidate targets from the 14 active compounds were collected from the TCMSP database. After eliminating the overlapping targets, 195 related targets were identified (Table 4 and Supplementary Table 1).

**Table 4:** Ten related targets of 195 targets list

No.	Gene Name	Protein Name
1	NOS2	Nitric oxide synthase 2
2	PTGS1	Cyclooxygenase-1
3	AR	Androgen receptor
4	PPARG	Peroxisome proliferator-activated receptor
5	PTGS2	Prostaglandin G/H synthase 2
6	HSP90AB1	Heat shock protein HSP 90-beta
7	PIK3CG	PI3-kinase subunit gamma
8	PRKACA	cAMP-dependent protein kinase catalytic subunit alpha
9	NCOA2	Nuclear receptor coactivator 2
10	DPP4	Dipeptidyl peptidase 4

### Choosing of the candidate targets of PCOS

**Fig. 3:**
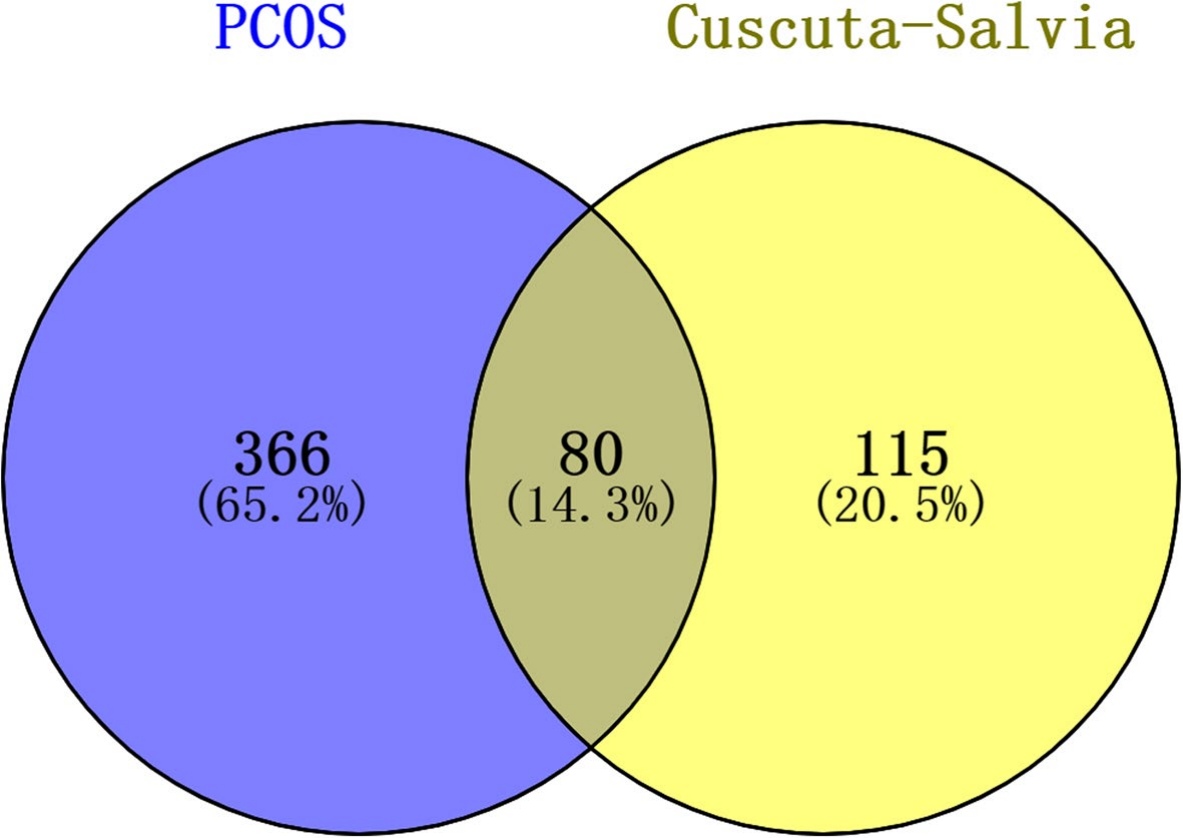
Venn diagram was applied to present all candidate targets of *Cuscuta-Salvia* and PCOS

To search for candidate targets related to PCOS, we used “polycystic ovary syndrome” as index keywords to identify 446 potential targets by removing duplicate values from the databases of DisGeNET and Genecard (relevance score ≥ 5). Eighty common targets between the targets of *Cuscuta-Salvia* (195 potential targets) and those of PCOS were collected using a Venn diagram (Fig. 3).

### Construction of an herb compound-PCOS target network

**Fig. 4:**
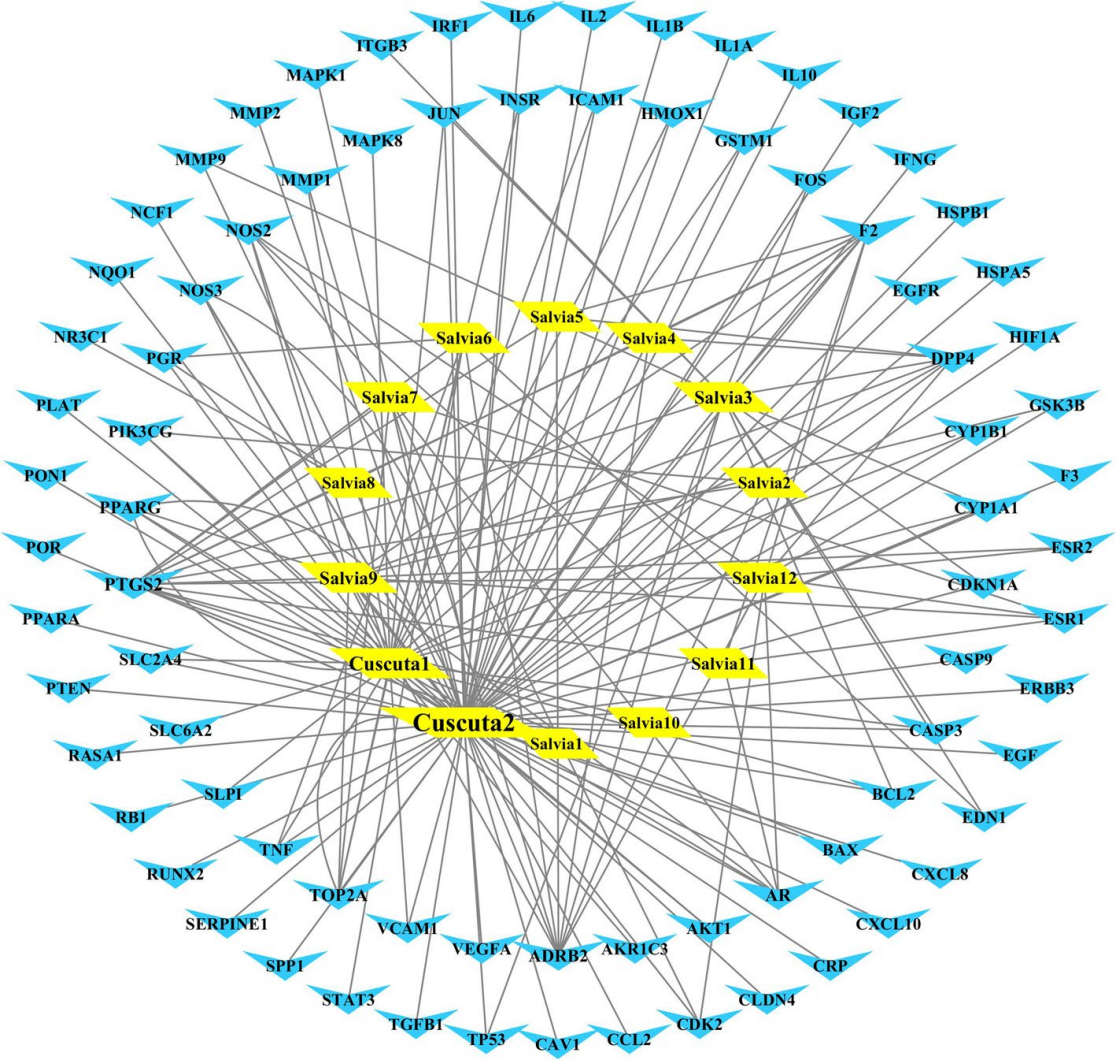
The network of herb compound-PCOS target in the treatment of PCOS

To determine the interaction between herbal compounds and PCOS targets, the active components and common targets were input into Cytoscape software to create a diagram of the herb compound-PCOS target network (Fig. 4). The top five active components are presented in Table 5 according to the degree values (the higher the degree values, the closer the relationship between the compound and the targets).

**Table 5:** The top 5 of active components of herb compound-PCOS target network

NO.	Molecular Name	Degree	Closenessc Cntrality
*Cuscuta* 2	quercetin	69	0.69402985
*Cuscuta* 1	kaempferol	30	0.44711538
*Salvia* 3	tanshinone iia	14	0.3907563
*Salvia* 9	dan-shexinkum d	12	0.38429752
*Salvia* 12	Isotanshinone II	10	0.37804878

### PPI network and core genes of disease-drug targets

**Fig. 5:**
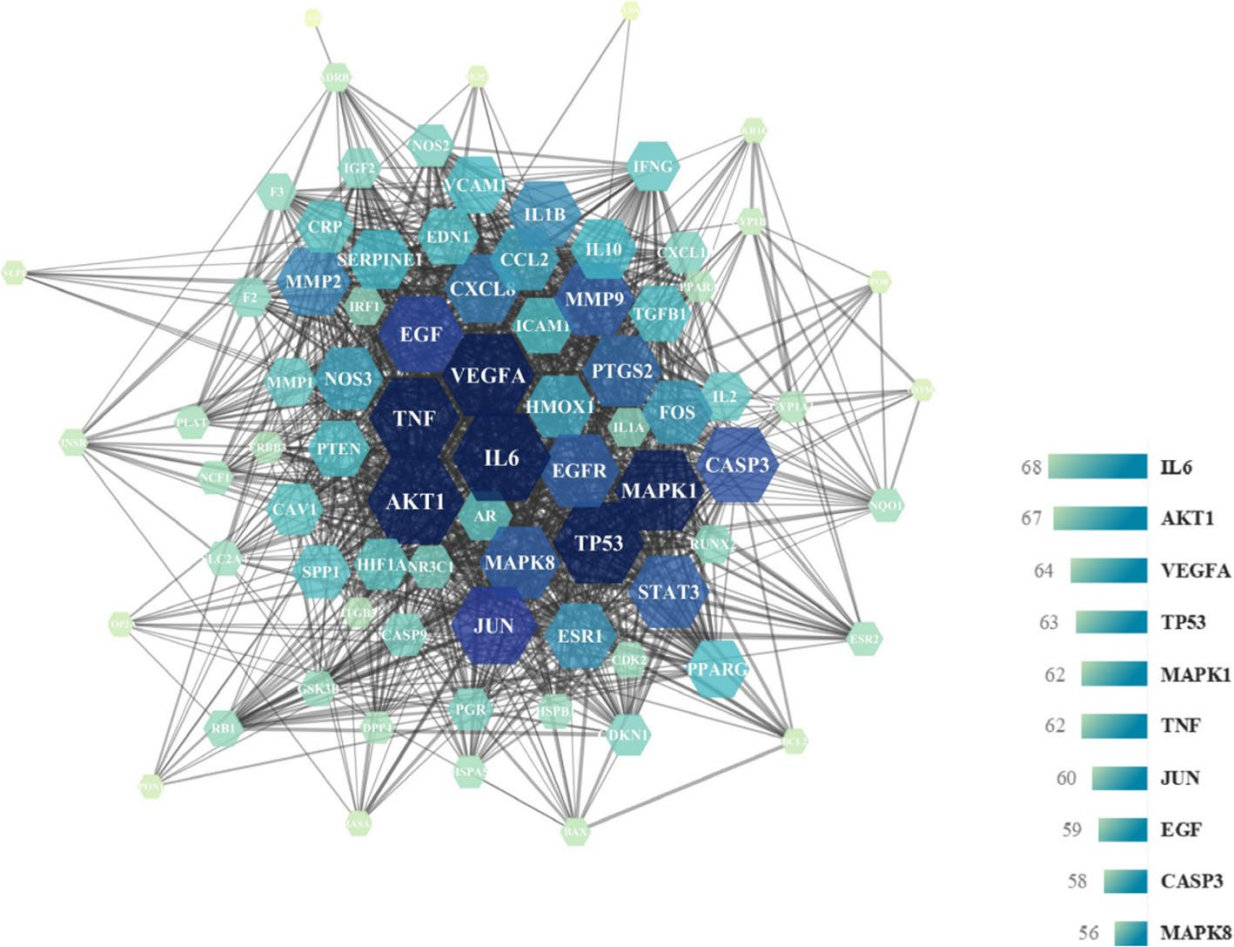
PPI network diagram of core targets. PPI network was drawn using STRING database

**Fig. 6:**
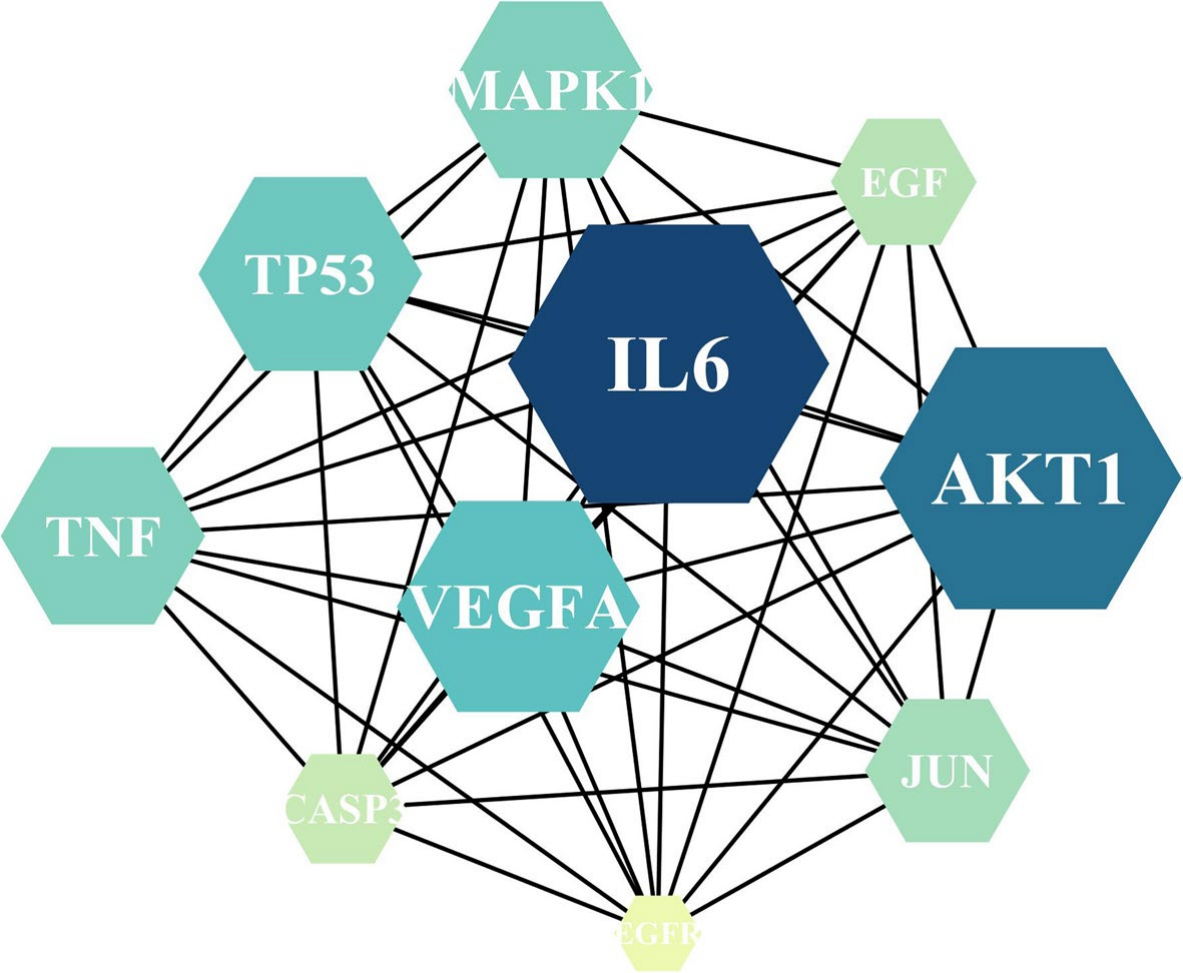
Network diagram of core targets of *Cuscuta-Salvia* against PCOS. PPI network was calculated and analyzed using Cytohubba plugin in Cytoscape v 3.7.2

We utilised the STRING database to establish a *Cuscuta-Salvia* target network and PCOS target networks, and the PPI network was visualised using Cytoscape v 3.7.2 software, which included 80 nodes and 1350 edges (Fig. 5). In addition, according to the degree ranking of nodes in STRING, the top 20 core targets were screened out (the higher the degree ranking, the closer the relationship between the proteins). Simultaneously, the PPI network was calculated and analysed using Cytohubba plugin in Cytoscape v3.7.2. As illustrated in Fig. 6 and Table 6, there were a total of 10 nodes and 45 edges in the PPI network. The 10 nodes included IL6, AKT1, VEGFA, TP53, TNF, MAPK1, JUN, EGF, CASP3, and EGFR.

**Table 6:** Protein target information

Gene name	Protein name	Degree	Closeness Centrality	Clustering Coefficient
IL6	Interleukin 6	68	0.87777778	0.51448639
AKT1	RAC-alpha serine/threonine-protein kinase	67	0.86813187	0.52464948
VEGFA	Vascular endothelial growth factor A	64	0.83157895	0.56597222
TP53	Cellular tumor antigen p53	63	0.82291667	0.5483871
MAPK1	Mitogen-activated protein kinase 1	62	0.81443299	0.54680063
TNF	Tumor necrosis factor	62	0.82291667	0.57588577
JUN	Transcription factor AP-1	60	0.7979798	0.58248588
EGF	Pro-epidermal growth factor	59	0.7979798	0.58036236
CASP3	Caspase-3	58	0.78217822	0.61766485
MAPK8	Mitogen-activated protein kinase 8	56	0.76699029	0.63181818

### GO functional enrichment analysis and KEGG pathway enrichment analysis

**Fig. 7:**
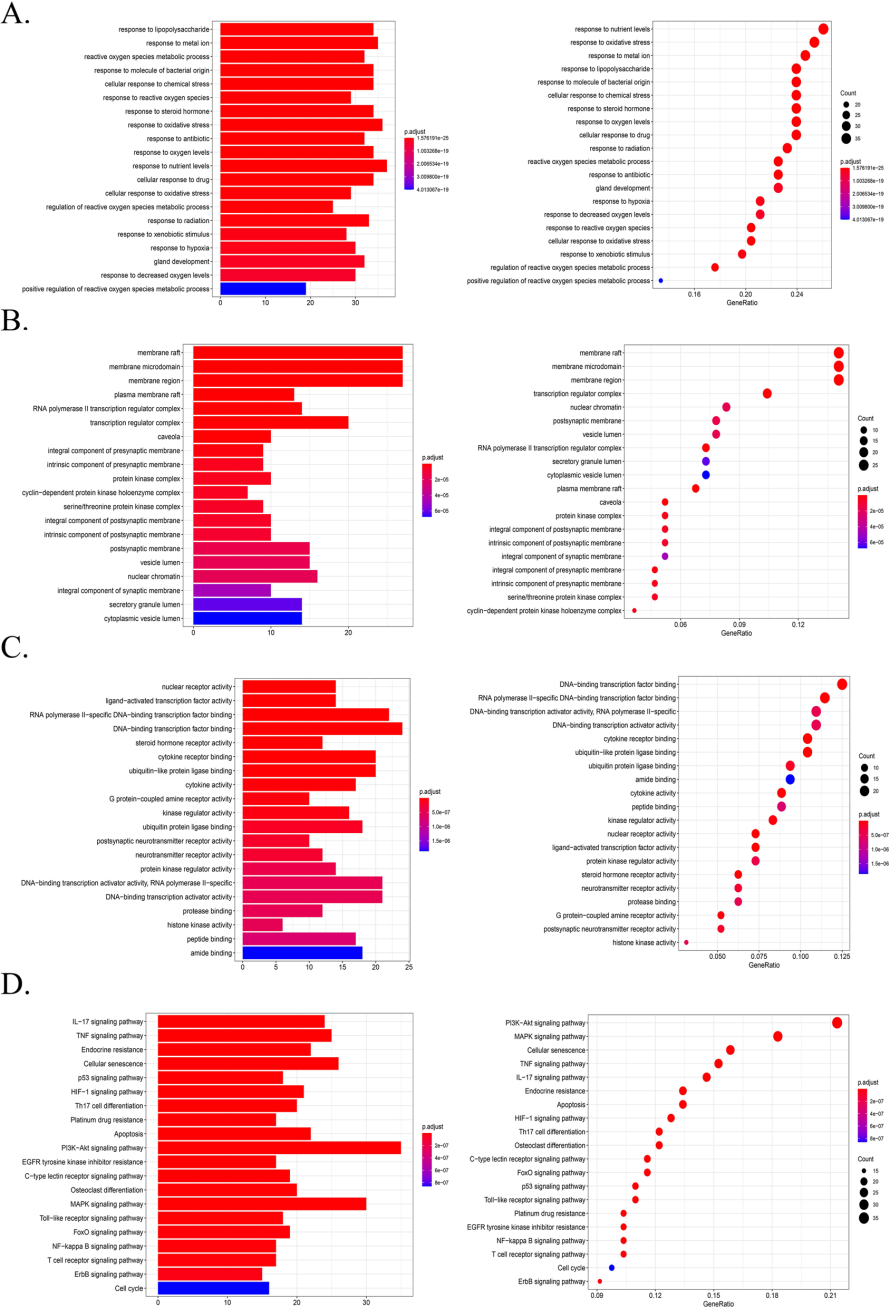
Representative diagram of GO function analysis and KEGG pathway analysis. **A**, **B**, and **C** Left, histogram of the biological processes (BP), the cellular component (CC), and the molecular function (MF) of *Cuscuta-Salvia* in the treatment of PCOS, respectively. **A**, **B**, and **C** Right, bubble chart of the BP, CC, and MF of *Cuscuta-Salvia* in the treatment of PCOS, respectively. **D** Left, histogram of KEEG pathways of *Cuscuta-Salvia* in the treatment of PCOS; Right, bubble chart of KEEG pathways of *Cuscuta-Salvia* in the treatment of PCOS

To analyse the biological characteristics of the predicted targets of *Cuscuta-Salvia* on PCOS in detail, GO and KEGG enrichment analyses were conducted using the “pathview” package in *R*. The GO terms included BP, CC, and MF terms. BP terms were mainly present in cellular responses to drugs, oxygen levels, response lipopolysaccharides, and molecules of bacterial origin (Fig. 7A). CC terms were mainly found in the membrane, transcription regulator complex, nuclear chromatin, postsynaptic membrane, and vesicle lumen (Fig. 7B). As shown in Fig. 7C, MF terms were mainly present in DNA-binding transcription factor binding, RNA polymerase II-specific DNA-binding transcription factor binding, DNA-binding transcription activator activity, RNA polymerase II-specific, DNA-binding transcription activator activity, and cytokine receptor binding. These factors can exert therapeutic effects on PCOS.

To investigate the underlying pathways of *Cuscuta-Salvia* in PCOS, a KEGG enrichment pathway analysis was performed. The filter was also set as an adjusted *P*-value < 0.05 and q-value < 0.05. As shown in Fig. 7D, the KEGG pathways were significantly enriched. The results of the KEGG enrichment analysis mainly involved the PI3K-Akt, MAPK, TNF, and IL-17 signalling pathways as well as cellular senescence, among others.

### Effect of *Cuscuta-Salvia* on the body weight of PCOS mice

**Fig. 8:**
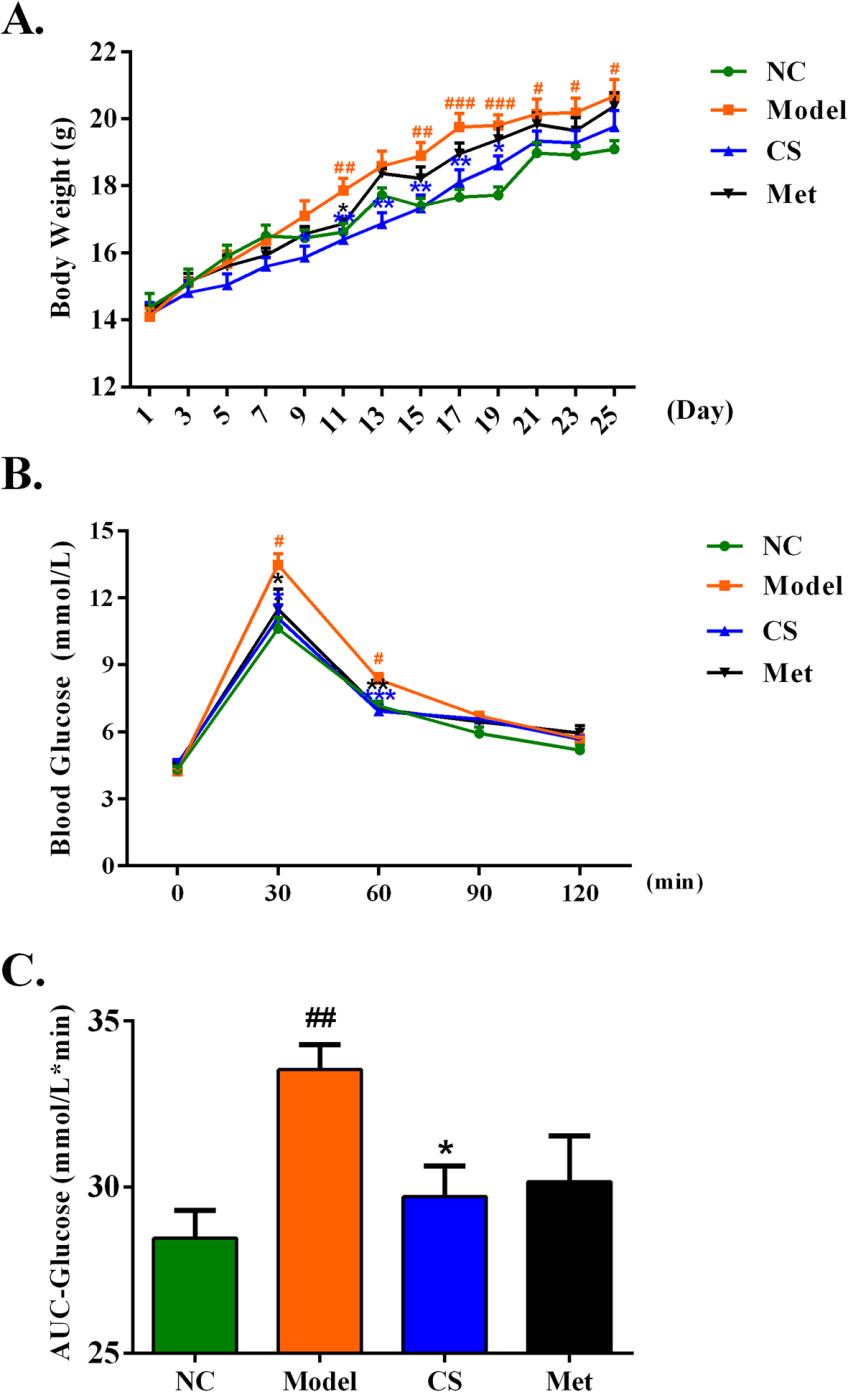
Assessment of body weight (**A**), blood glucose levels (**B**), and statistical chart of OGTT (**C**) in PCOS mice. Results are presented as means± SEM, ^##^*P* < 0.01vs. the NC group; ^*^P < 0.05 vs. the model group

To determine whether there was an increase in body weight in the letrozole-induced PCOS mouse model [56], mouse weight was measured. As shown in Fig. 8A, compared with that in the NC group, the weight of the mice in the Model group increased significantly after day 15. Compared that in the Model group, the weight of the mice in the CS and Met groups decreased; the weight of the mice in the CS group was greater than that of the mice in the Met group.

### OGTT results in PCOS mice

An OGTT was conducted to examine glucose tolerance in the letrozole-induced PCOS mouse model. Compared with that in the NC group, the AUC of the model group increased significantly (*P* < 0.01). Compared with that in the model group, the AUC of the CS and Met groups decreased (*P* < 0.05). Moreover, there were no differences in the AUC of blood glucose levels between the CS and Met groups (Fig. 8B-C).

### Histological changes in ovary, liver, and adipose tissues

**Fig. 9:**
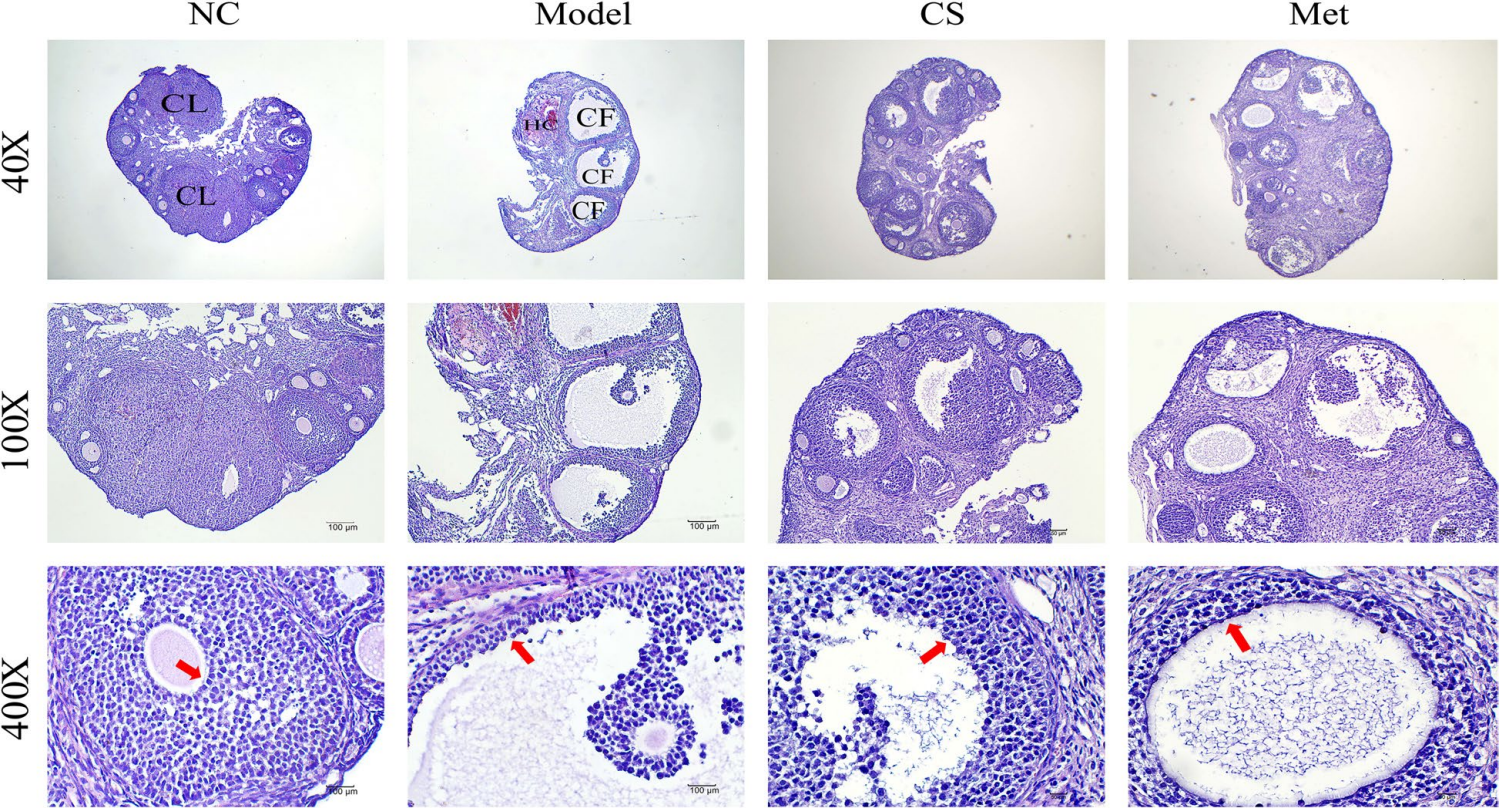
Assessment of histopathological change on *Cuscuta-Salvia* on ovary tissue in PCOS mice on day 30 (40×, 100×, 400×). Red arrows indicate granulosa layer

As shown in Fig. 9, in mice ovary tissues, the NC group mice had many follicles at various stages of development at the site of the peripheral cortex. We observed normal follicular developmental synchronisation, stromal tissue, and normal morphology. In the model group, numerous antral follicles were observed. We also observed incompact follicles, increased medullary area, and enlarged vessel networks. In addition, cystic follicles and haemorrhagic cystswere observed. In the CS and Met groups, the morphology in ovaries was more neatly arranged than that in the model group, and the number of antral follicles was reduced. Meanwhile, the CS group mice had a more neatly arranged ovarian morphology than that of the Met group mice.

**Fig. 10:**
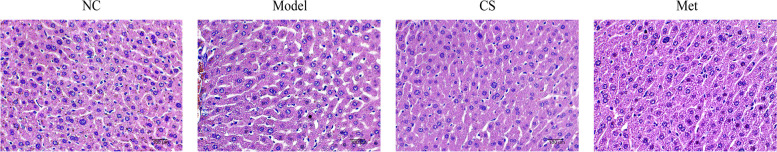
Assessment of histopathological change on *Cuscuta-Salvia* on liver tissue in PCOS mice on day 30 (400×). We observed histopathological changes

As shown in Fig. 10, the mouse liver tissues, there were more neutrophils in model group than that in the NC group and a normal liver architecture. Compared with that in the model group, the CS and Met groups results showed that the number of neutrophils significant reduced and vacuolar degenerated.

**Fig. 11:**
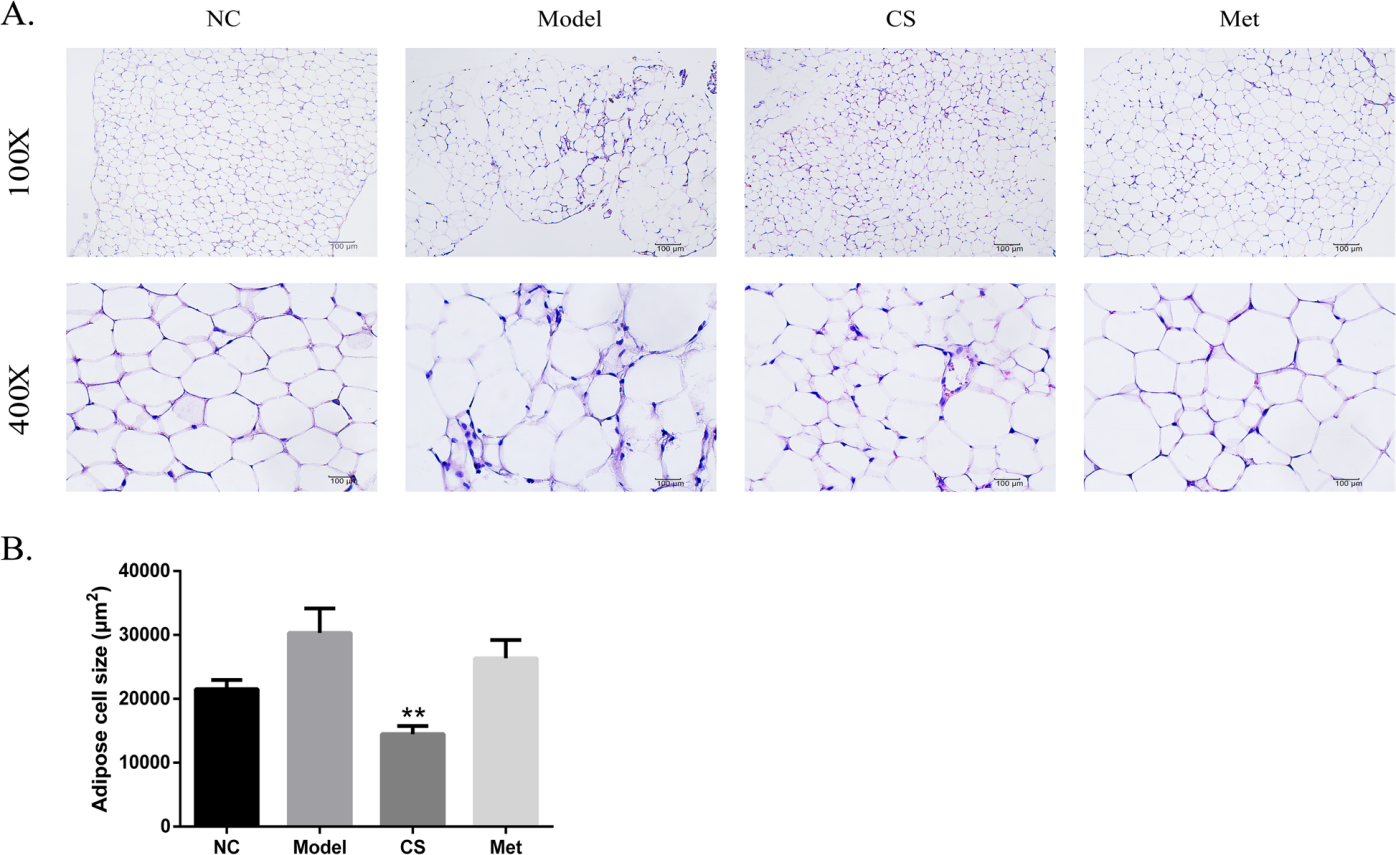
Assessment of histopathological change on *Cuscuta-Salvia* on adipose tissue in PCOS mice on day 30 (100×, 400×). **A** Representative photography of adipocyte. **B** Quantification of adipocyte cell size using the Image J software (*n* = 6). Results are presented as means± SEM, ^##^*P* < 0.01vs. the NC group; ^**^P < 0.01 vs. the model group

As shown in Fig. 11, in the mouse adipose tissue, there were enhanced irregular abdominal adipocytes and inflammatory infiltration in the model group. Compared with the model group, the CS and Met groups results showed significantly improved abdominal adipocyte and inflammatory infiltration. In addition, the average size of adipocytes in the PCOS group was the greatest among the groups, with the lowest size in the CS group (*P* < 0.01).

### Effect of *Cuscuta-Salvia* on the mRNA expression of AR, LHb, FSHb, CYP17a1, CYP19a1, IL6, AKT1, VEGFA, TP53, MAPK1, JUN, and EGF in the ovarian tissues of PCOS mice

**Fig. 12:**
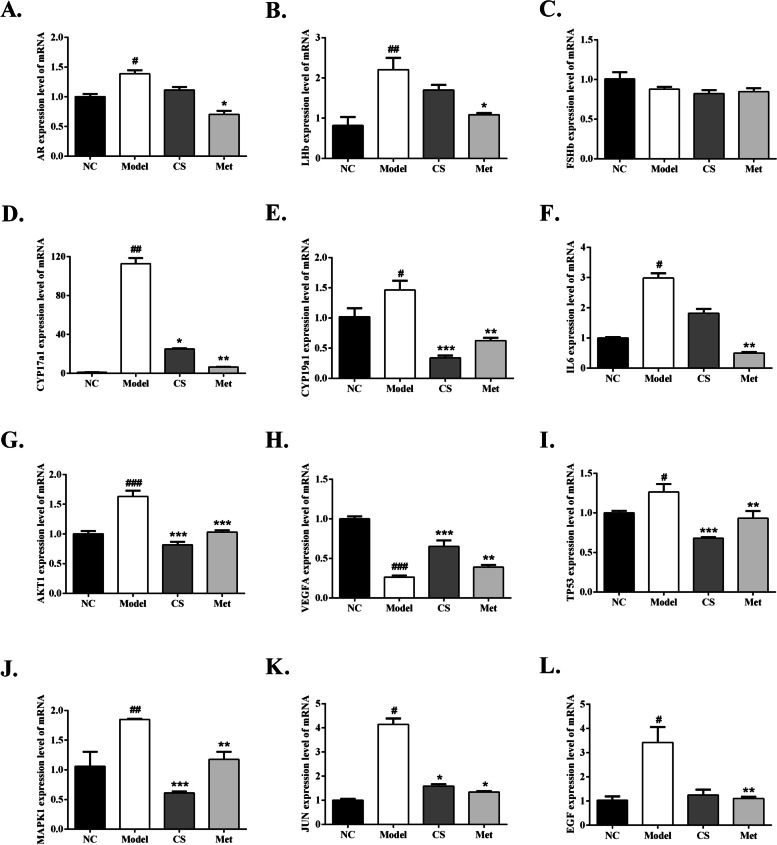
Effects of *Cuscuta-Salvia* (CS) administration on **A** AR, **B** LHb, **C** FSHb, **D** CYP17a1, **E** CYP19a1, **F **IL6, **G** AKT1, **H** VEGFA, **I** TP53, **J** MAPK1, **K** JUN, **L** EGF. All data are expression as mean ± standard error of mean ($\overline{x}$ ±SEM) (*n* = 3). ^###^*P* < 0.001, ^##^*P* < 0.01, and ^#^*P* < 0.05 vs. the normal control group; ^***^P < 0.001, ^**^P < 0.01, and ^*^P < 0.05 vs. the model group

As shown in Fig. 12, compared with that in the NC group, the mRNA expression of AR, LHb, and CYP17a1 increased significantly in the model group (*P* < 0.01 or *P* < 0.05). Compared with that in the model group, the mRNA expression of CYP17a1 decreased significantly in the CS and Met groups (*P* < 0.01 or *P* < 0.05). There were no differences in the mRNA expression of AR and LHb in the CS group, although it decreased compared to that in the model group. Compared with that in the NC group, the mRNA expression of CYP19a1 increased significantly in the model group (*P* < 0.05). Compared with that in the model group, the mRNA expression of CYP19a1 significantly decreased in the CS and Met groups (*P* < 0.001 or *P* < 0.01). Compared with that in the NC group, the mRNA expression of FSHb decreased in the model group. Compared with that in the model group, the mRNA expressions were no differences in the CS and Met groups.

qRT-PCR was used to examine the mRNA expression of IL6, AKT1, VEGFA, TP53, MAPK1, JUN, and EGF in mouse ovarian tissue. As shown in Fig. 12, the mRNA expression of IL6, JUN, and EGF was significantly higher in the model group than that in the NC group (*P* < 0.05). Compared with that in the model group, the mRNA expression of IL6 and EGF in the CS group decreased, while the mRNA expression of JUN in the CS group decreased significantly (*P* < 0.05). Furthermore, compared with that in the model group, the mRNA expressions of IL6, JUN, and EGF significantly decreased in the Met groups. (*P* < 0.01 or *P* < 0.05).

As shown in Fig. 12, compared with that in the NC group, the mRNA expression of AKT1 and TP53 and MAPK1 increased significantly in the model group (*P* < 0.001 or *P* < 0.01 or *P* < 0.05). Compared to that in the model group, the mRNA expression of Akt1, TP53, and MAPK1 decreased markedly in the CS and Met groups (*P* < 0.001 or *P* < 0.01). Compared with that in the NC group, the mRNA expression of VEGFA decreased significantly in the model group (*P* < 0.001). Compared with that in the model group, the mRNA expression of VEGFA significantly increased in the CS and Met groups (*P* < 0.001 or *P* < 0.01).

### Effect of *Cuscuta-Salvia* on the ovarian tissue protein expression of TP53, p-AKT, AKT, p-MAPK, MAPK, c-JUN, and VEGFA in PCOS mice

**Fig. 13:**
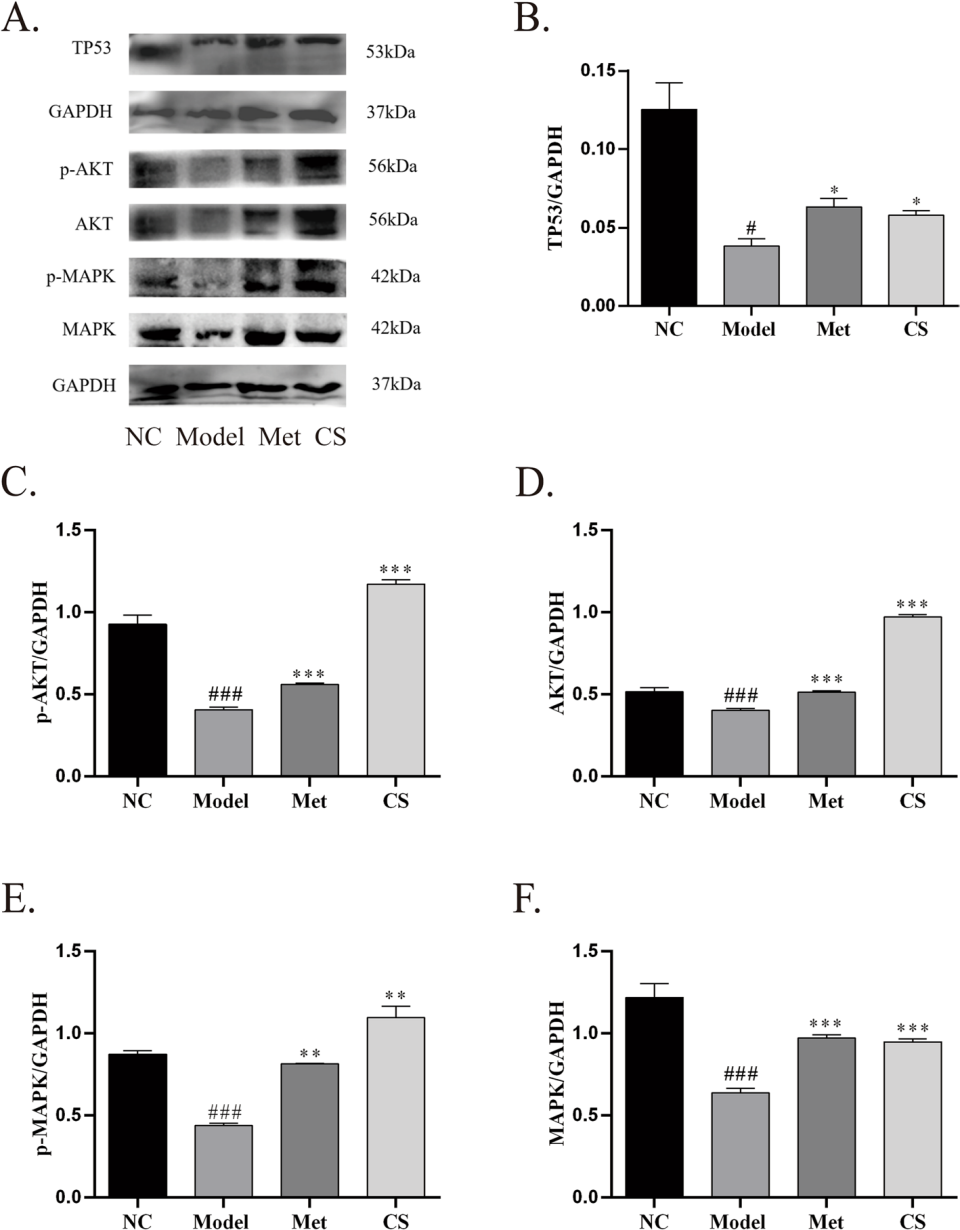
Effects of *Cuscuta-Salvia* (CS) administration on (**A**): Representative Western blot of TP53, p-AKT, AKT, p-MAPK, and MAPK. **B C D E F**: Representative data of TP53, p-AKT/GAPDH, AKT/GAPDH, p-MAPK/GAPDH, and MAPK/GAPDH. All data are expression as mean ± standard error of mean ($\overline{x}$ ±SEM) (*n* = 3). ^###^*P* < 0.001, ^##^*P* < 0.01, and ^#^*P* < 0.05 vs. the normal control group; ^***^P < 0.001, ^**^P < 0.01, and ^*^P < 0.05 vs. the model group

As shown in Fig. 13, compared to that in the NC group, the expression of TP53, p-AKT, AKT, p-MAPK, and MAPK decreased significantly in the model group (*P* < 0.001 or *P* < 0.05). Relative to that in the model group, the expression of TP53, p-AKT, AKT, p-MAPK, and MAPK increased significantly in the CS and Met groups (*P* < 0.001, *P* < 0.01*,* or *P* < 0.05).

**Fig. 14:**
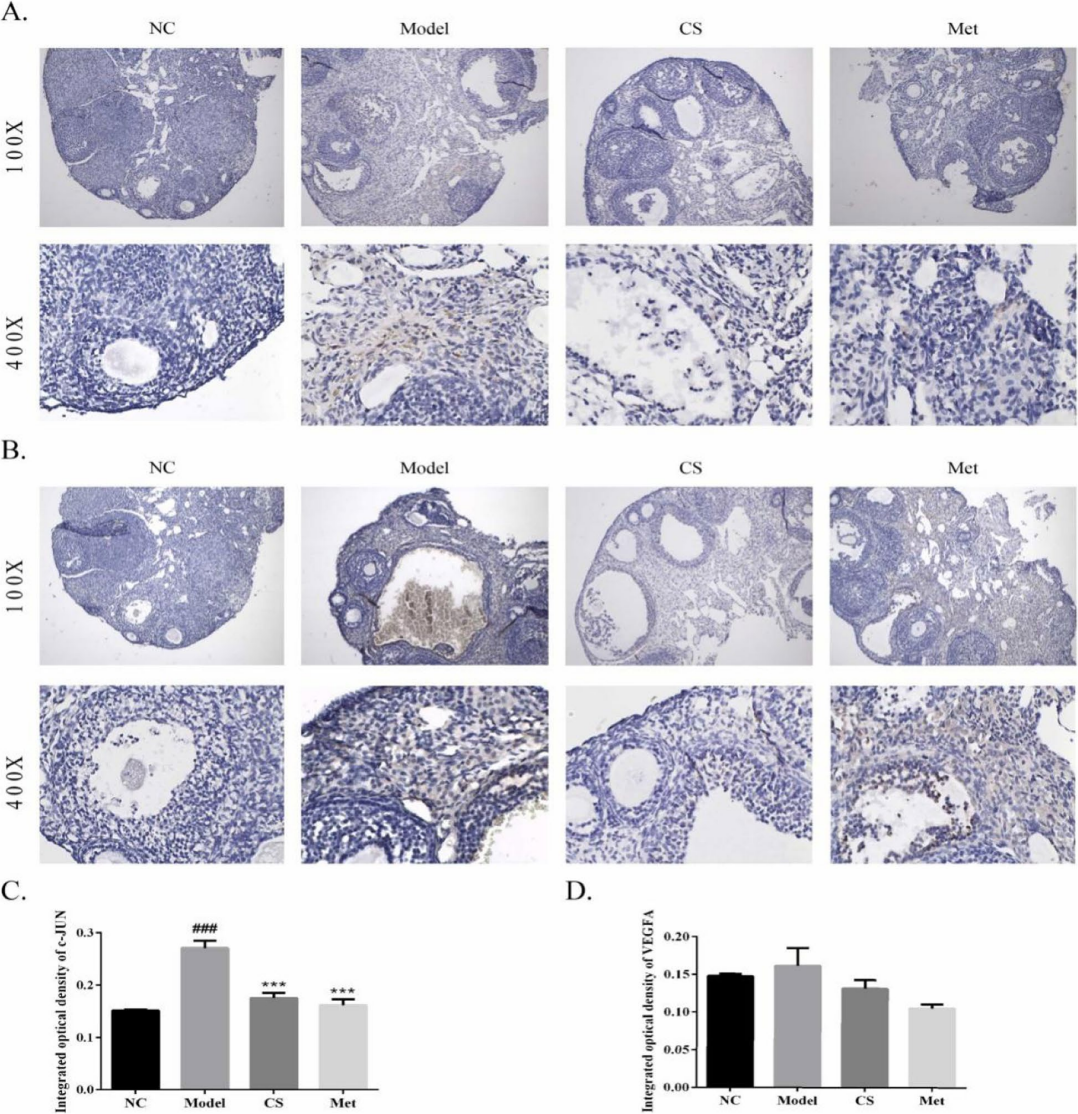
Effects of *Cuscuta-Salvia* (CS) administration on c-JUN and VEGFA. **A B** Representative photography of c-JUN and VEGFA, respectively (100× and 400× magnification). **C D** Semi-quantitative scores of the expression of c-JUN and VEGFA. All data are expression as mean ± standard error of mean ($\overline{x}$ ±SEM) (*n* = 3). ^###^*P* < 0.001, ^##^*P* < 0.01, and ^#^*P* < 0.05 vs. the normal control group; ^***^P < 0.001, ^**^P < 0.01, and ^*^P < 0.05 vs. the model group

As shown in Fig. 14, the c-JUN and VEGFA levels were determined using immunohistochemistry. Compared with that in the NC group, the expression of c-JUN and VEGFA in the model group increased (*P* < 0.001). Relative to that in the model group, the expression of c-JUN and VEGFA decreased in the CS and Met groups (*P* < 0.001).

## Discussion

The global incidence of PCOS is higher during the reproductive age. Metformin is a good insulin sensitizer and can reduce weight. It is not known whether metformin is effective in the treatment of Non-obese women. It should be used with caution in non-obese PCOS patients because of the gastrointestinal adverse reactions. TCM treatment is a very effective method, but more basic research and a large amount of data are needed to prove it [34]. And an increasing number of TCM formulas have been widely used to treat several diseases owing to their multiple targets. In this study, we compared the effect of a TCM formula on PCOS with that of an insulin sensitiser. In addition, we utilised network pharmacology to explore the material basis and underlying molecular mechanisms of *Cuscuta-Salvia* in the treatment of PCOS.

First, we identified the components of *Cuscuta-Salvia* using UHPLC-ESI-Q-TOF-MS. Next, we selected the active components and targets of *Cuscuta-Salvia* and a network of TCM components and targets was established. Based on the network diagram of the herb-compound target-PCOS target, we found that quercetin and kaempferol were the main active compounds with higher degree values. Studies on the use of quercetin against PCOS have suggested that in clinical trials or animal experiments, quercetin mainly improves lipid abnormalities, enhances sex hormone levels, reduces insulin resistance, and increases anti-inflammation [22, 37, 46]. Previous studies have reported that quercetin plays a role in insulin resistance, insulin tolerance, and glucose tolerance in type 2 diabetes mellitus, as well as other metabolism-related diseases in rodents [4]. Kaempferol enhances Akt expression and hexokinase activity in the liver, which increases glucose intake and its metabolism, improves blood sugar levels, and enhances insulin sensitivity [2]. Additionally, kaempferol decreases lipid stores induced by palmitic acid, endoplasmic reticulum stress, and pancreatic β cell dysfunction via AMPK/mTOR pathway-mediated lipophagocytosis [42]. In our experiments, we found that *Cuscuta-Salvia* could reduce mice weight gain and glucose tolerance (Fig. 8).

Next, we identified 80 common targets, which might be targets for *Cuscuta-Salvia* in the treatment of PCOS. Based on the degree values obtained by Cytoscape v3.7.2 software, 20 core targets were screened from the 80 common targets. The Cytohubba plugin allowed us to obtain 10 core targets were obtained from the 20 core targets (IL6, AKT1, VEGFA, TP53, TNF, MAPK1, JUN, EGF, CASP3, and EGFR).

The results of our study showed that the mRNA levels of IL6, AKT1, TP53, MAPK1, JUN, and EGF were higher in the model group than those in the normal control group; however, these levels decreased in the treatment groups. The mRNA level of VEGFA was lower in the model group than that in the normal control group; however, in the treatment groups, the VEGFA mRNA levels increased. In PCOS patients, IL6 is recognised as an early chronic inflammatory marker [49]. In addition, IL6 is secreted in visceral adipose tissue in PCOS group [12]. Patients with hyperandrogenic PCOS have higher AKT1 levels [27]. VEGFA medicates PCOS formation [5]. TP53 levels can decrease in PCOS patients [44], while the MAPK1 levels are higher, leading to insulin resistance [16]. PCOS mice have been rescued by regulating the JUN pathway [29]. EGF overexpression has been measured in PCOS [3]. CASP3 plays a critical role in initiating apoptosis [7]. EGFR participates in the autophagy activation in PCOS [24].

Then, we identified many pathways, including the PI3K-Akt, MAPK, TNF, and IL-17 signalling pathways as well as cellular senescence. According to the literature, the activated PI3K-Akt signalling pathway can increase insulin sensitivity and regulate androgen levels [38, 48]. Suppression of the MAPK signalling pathway can reduce PCOS pathology [55]. In the meantime, MAPK regulates the induction and progression of EMT, while EMT participates in certain biological functions, such as uterine development, reproductive disorders, and so on [17]. Cellular senescence is a normal process owing to the formation of oxygen free radicals, and is associated with telomere length; prolonged telomere lengths can reverse PCOS [43]. TNF is associated with an enhanced susceptibility to PCOS [27], and androgen levels are affected by TNF-α [23]. In patients with PCOS, increased IL-17 levels can lead to the formation of an inflammatory response [41]. A previous study have reported that higher serum IL-17 levels are associated with PCOS aetiology [1].

Finally, the HE staining results showed that in the PCOS model, *Cuscuta-Salvia* administration could improve tissue morphology and treat PCOS. We selected the core targets for qRT-PCR validation and confirmed that this TCM did improve the disease. In addition, the mRNA levels of AR, LHR, CYP17a1, and CYP19a1 were higher in the PCOS mice than those in the normal animals. In the CS and Met groups, the mRNA levels of AR, LHR, CYP17a1, and CYP19a1 decreased compared to those in the NC group. Additionally, the mRNA levels of FSHb were lower in the PCOS mice than those in the normal mice. After the treatment, the FSHb mRNA levels decreased. High AR expression is observed in patients with PCOS [25]. Many patients with PCOS have elevated LHb levels, [6], and FHSb is associated with LHb levels [35]. In addition, CYP17a1 protein is expressed in women with PCOS [14] and CYP19a1 mRNA levels are high in rats with PCOS rats [18]. These results are consistent with those of the present study.

To sum up, in this study we found 10 core targets and showed that *Cuscuta-Salvia* improved PCOS pathology. Thus, these targets are mechanistically important for PCOS and may exert a positive effect on its treatment. This study provides novel insights into the perspectives and challenges of the future studies.

## Conclusion

In summary, the results of the present study, based on a combination of network pharmacology and animal experiments, demonstrate that *Cuscuta-Salvia* exerted its pharmacological effects on PCOS. Firstly, the main components of *Cuscuta-Salvia* were identified by UHPLC and screened using the TCMSP database. Next, 80 common targets and 20 pathways were identified. Finally, the core genes were tested using in vivo experiments.

In conclusion, this approach was confirmed to be quick and effective. In addition, our findings can provide a reference for similar research in the future.

## Supplementary Information

**Additional file 1.**

**Additional file 2.**

## Data Availability

The raw data supporting the conclusions of this manuscript will be made available by the authors, without undue reservation, to any qualified research.
