# Supplementary material for: Investigation of the mechanisms and experimental verification of Cuscuta-Salvia in the treatment of polycystic ovary syndrome (PCOS) via network pharmacology
Source: J Ovarian Res. 2022 Apr 4;15:40. doi: 10.1186/s13048-022-00964-8 (PMC8978390; doi:10.1186/s13048-022-00964-8)
Supplement: Supplementary file 1 — Additional file 1. [file 13048_2022_964_MOESM1_ESM.docx]

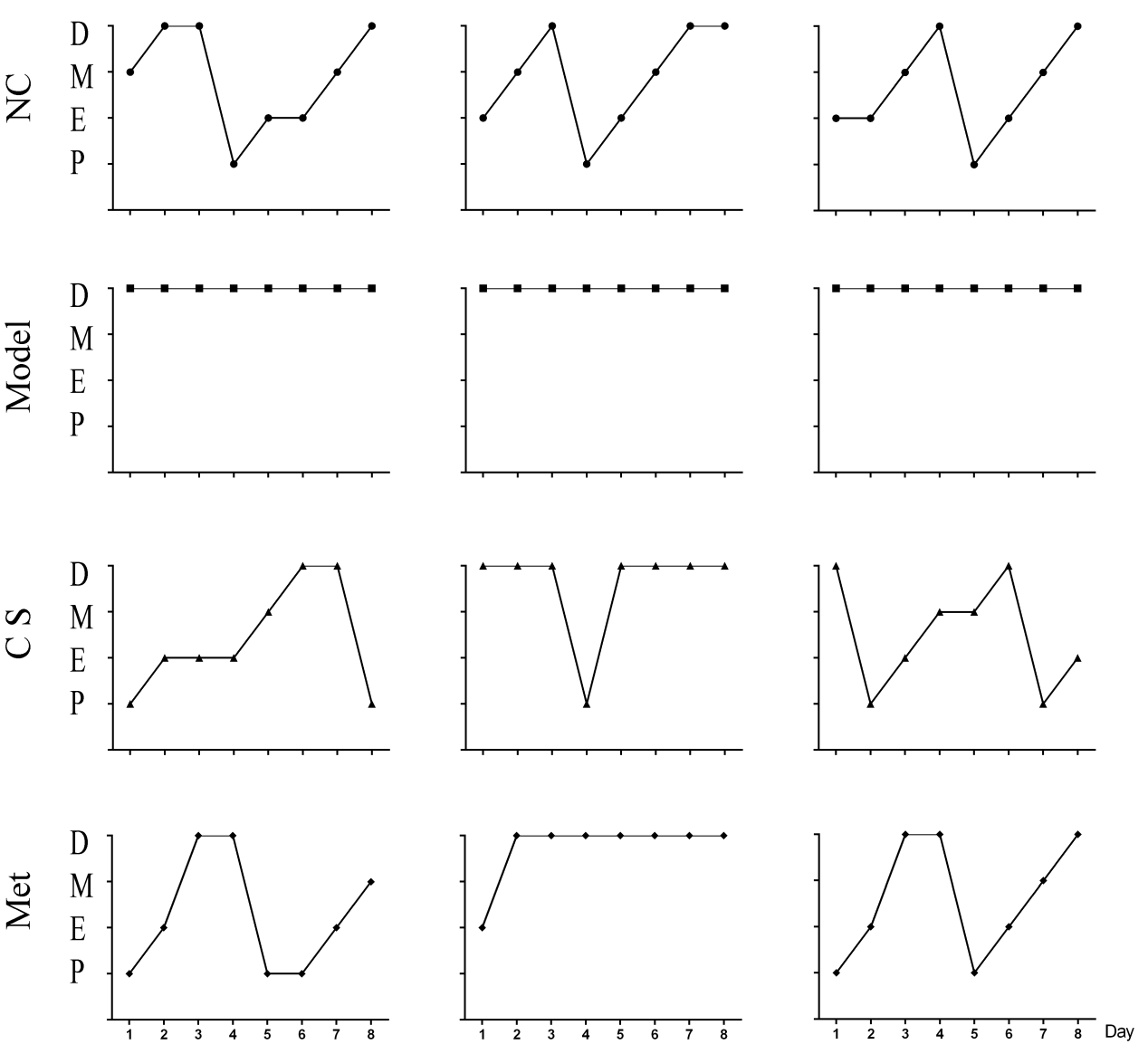


Supplementary figure 1. Estrous cycle of the four groups (n=3, from day 17 to 25, monitored for eight consecutive days; M: metestrus, E: estrus, P: proestrus, D: diestrus).
