# Supplementary material for: Investigation of the mechanisms and experimental verification of Cuscuta-Salvia in the treatment of polycystic ovary syndrome (PCOS) via network pharmacology
Source: J Ovarian Res. 2022 Apr 4;15:40. doi: 10.1186/s13048-022-00964-8 (PMC8978390; doi:10.1186/s13048-022-00964-8)
Supplement: Supplementary file 2 — Additional file 2. [file 13048_2022_964_MOESM2_ESM.docx]

Supplementary table 1.195 related targets list.

| No. | Gene Name | Protein Name |
| --- | --- | --- |
| 1 | NOS2 | Nitric oxide synthase 2 |
| 2 | PTGS1 | Cyclooxygenase-1 |
| 3 | AR | Androgen receptor |
| 4 | PPARG | Peroxisome proliferator-activated receptor |
| 5 | PTGS2 | Prostaglandin G/H synthase 2 |
| 6 | HSP90AB1 | Heat shock protein HSP 90-beta |
| 7 | PIK3CG | PI3-kinase subunit gamma |
| 8 | PRKACA | cAMP-dependent protein kinase catalytic subunit alpha |
| 9 | NCOA2 | Nuclear receptor coactivator 2 |
| 10 | DPP4 | Dipeptidyl peptidase 4 |
| 11 | PRSS1 | Protease serine 1 |
| 12 | PGR | Progesterone receptor |
| 13 | F2 | Coagulation factor II |
| 14 | CHRM1 | Muscarinic acetylcholine receptor M1 |
| 15 | NOS3 | Nitric oxide synthase 3 |
| 16 | GABRA2 | Gamma-aminobutyric acid receptor subunit alpha-2 |
| 17 | ACHE | Acetylcholinesterase |
| 18 | SLC6A2 | Sodium-dependent noradrenaline transporter |
| 19 | CHRM2 | Muscarinic acetylcholine receptor M2 |
| 20 | ADRA1B | Alpha-1B adrenergic receptor |
| 21 | GABRA1 | Gamma-aminobutyric acid receptor subunit alpha-1 |
| 22 | TOP2A | DNA topoisomerase 2-alpha |
| 23 | F7 | Coagulation factor VII |
| 24 | RELA | Transcription factor p65 |
| 25 | IKBKB | Inhibitor of nuclear factor kappa-B kinase subunit beta |
| 26 | AKT1 | Threonine-protein kinase |
| 27 | BCL2 | Apoptosis regulator BCL-2 |
| 28 | BAX | Apoptosis regulator BAX |
| 29 | TNF | Tumor necrosis factor |
| 30 | JUN | Transcription factor AP-1 |
| 31 | AHSA1 | Activator of 90 kDa heat shock protein ATPase homolog 1 |
| 32 | CASP3 | Caspase-3 |
| 33 | MAPK8 | Mitogen-activated protein kinase 8 |
| 34 | XDH | Xanthine dehydrogenase |
| 35 | MMP1 | Matrix metalloproteinase-1 |
| 36 | STAT1 | Signal transducer and activator of transcription 1 |
| 37 | CDK1 | Cyclin-dependent kinase 1 |
| 38 | HMOX1 | Heme oxygenase 1 |
| 39 | CYP3A4 | Cytochrome P450 3A4 |
| 40 | CYP1A2 | Cytochrome P450 1A2 |
| 41 | CYP1A1 | Cytochrome P450 1A1 |
| 42 | ICAM1 | Intercellular adhesion molecule 1 |
| 43 | SELE | E-selectin |
| 44 | VCAM1 | Vascular cell adhesion protein 1 |
| 45 | NR1I2 | Nuclear receptor subfamily 1 group I member 2 |
| 46 | CYP1B1 | Cytochrome P450 1B1 |
| 47 | ALOX5 | Polyunsaturated fatty acid 5-lipoxygenase |
| 48 | HAS2 | Hyaluronan synthase 2 |
| 49 | GSTP1 | Glutathione S-transferase 2 |
| 50 | AHR | Aryl hydrocarbon receptor |
| 51 | PSMD3 | 26S proteasome non-ATPase regulatory subunit 3 |
| 52 | SLC2A4 | Solute carrier family 2 member 4 |
| 53 | NR1I3 | Nuclear receptor subfamily 1 group I member 3 |
| 54 | INSR | Insulin receptor |
| 55 | DIO1 | Death-inducer obliterator 1 |
| 56 | PPP3CA | Calmodulin-dependent calcineurin A subunit alpha isoform |
| 57 | GSTM1 | Glutathione S-transferase Mu 1 |
| 58 | GSTM2 | Glutathione S-transferase Mu 2 |
| 59 | AKP1C3 | Aldo-keto reductase family I member C3 |
| 60 | SLPI | Antileukoproteinase |
| 61 | AKR1B1 | Aldo-keto reductase family 1 member B1 |
| 62 | KCMH2 | Potassium voltage gated channel subfamily H member 2 |
| 63 | SCN5A | Sodium channel protein type 5 subunit alpha |
| 64 | F10 | Coagulation factor X |
| 65 | ADRB2 | Beta-2 adrenergic receptor |
| 66 | MMP3 | Matrix metalloproteinase-3 |
| 67 | RXRA | Retinoic acid receptor RXR-alpha |
| 68 | MAOB | Amine oxidase |
| 69 | EGFR | Epidermal growth factor receptor |
| 70 | VEGFA | Vascular endothelial growth factor A |
| 71 | CCND1 | G1/S-specific cyclin-D1 |
| 72 | BCL2L1 | Bcl-2-like protein 1 |
| 73 | FOS | Proto-oncogene c-Fos |
| 74 | CDKN1A | Cyclin-dependent kinase inhibitor 1 |
| 75 | EIF6 | Eukaryotic translation initiation factor 6 |
| 76 | CASP9 | Caspase-9 |
| 77 | PLAU | Urokinase-type plasminogen activator |
| 78 | MMP2 | Matrix metalloproteinase-2 |
| 79 | MMP9 | Matrix metalloproteinase-9 |
| 80 | MAPK1 | Mitogen-activated protein kinase 1 |
| 81 | IL10 | Interleukin-10 receptor subunit alpha |
| 82 | EGF | Pro-epidermal growth factor |
| 83 | RB1 | Retinoblastoma-associated protein |
| 84 | IL6 | Interleukin-6 receptor subunit alpha |
| 85 | CDKN2A | Cyclin-dependent kinase inhibitor 2A |
| 86 | TP53 | Cellular tumor antigen p53 |
| 87 | ELK1 | ETS domain-containing protein Elk-1 |
| 88 | NFKBIA | NF-kappa-B inhibitor alpha |
| 89 | POR | NADPH-cytochrome P450 reductase |
| 90 | ODC1 | Ornithine decarboxylase |
| 91 | CASP8 | Caspase-8 |
| 92 | TOP1 | DNA topoisomerase 1 |
| 93 | RAF1 | RAF proto-oncogene serine/threonine-protein kinase |
| 94 | SOD1 | Superoxide dismutase [Cu-Zn] |
| 95 | PRKCA | Protein kinase C alpha type |
| 96 | HIF1A | Hypoxia-inducible factor 1-alpha |
| 97 | RUNX1T1 | Protein CBFA2T1 |
| 98 | CDK1 | Cyclin-dependent kinase inhibitor 1 B |
| 99 | HSPA5 | Endoplasmic reticulum chaperone BiP |
| 100 | ERBB2 | Receptor tyrosine-protein kinase erbB-2 |
| 101 | ACACA | Acetyl-CoA carboxylase 1 |
| 102 | CAV1 | Caveolin-1 |
| 103 | MYC | Myc proto-oncogene protein |
| 104 | F3 | Tissue factor |
| 105 | GJA1 | Gap junction alpha-1 protein |
| 106 | IL1B | Interleukin-1 beta |
| 107 | CCL2 | C-C motif chemokine 2 |
| 108 | PTGER3 | Prostaglandin E2 receptor EP3 subtype |
| 109 | CXCL8 | Interleukin-8 |
| 110 | PRKCB | Protein kinase C beta type |
| 111 | BIRC5 | Baculoviral IAP repeat-containing protein 5 |
| 112 | DUOX2 | Dual oxidase 2 |
| 113 | HSPB1 | Heat shock protein beta-1 |
| 114 | TGFB1 | Transforming growth factor beta-1 proprotein |
| 115 | SULT1E1 | Sulfotransferase 1E1 |
| 116 | MGAM | Maltase-glucoamylase |
| 117 | IL2 | Interleukin-2 |
| 118 | CCNB1 | G2/mitotic-specific cyclin-B1 |
| 119 | PLAT | Tissue-type plasminogen activator |
| 120 | THBD | Thrombomodulin |
| 121 | SERPINE1 | Plasminogen activator inhibitor 1 |
| 122 | COL1A1 | Collagen alpha-1 (I) chain |
| 123 | IFNG | Interferon gamma |
| 124 | ALOX5AP | Arachidonate 5-lipoxygenase-activating protein |
| 125 | PTEN | Phosphatidylinositol-3,4,5-trisphosphate 3-phosphatase |
| 126 | IL1A | Interleukin-1 beta |
| 127 | MPO | Myeloperoxidase |
| 128 | NCF1 | Neutrophil cytosol factor 1 |
| 129 | ABCG2 | Broad substrate specificity ATP-binding cassette transporter ABCG2 |
| 130 | NFE2L2 | Nuclear factor erythroid 2-related factor 2 |
| 131 | NQO1 | NAD (P) H dehydrogenase [quinone] 1 |
| 132 | PARP1 | Poly [ADP-ribose] polymerase 1 |
| 133 | COL3A1 | Collagen alpha-1 (III) chain |
| 134 | CXCL11 | C-X-C motif chemokine 11 |
| 135 | CXCL2 | C-X-C motif chemokine 2 |
| 136 | DCAF5 | DDB1-and CUL4-associated factor 5 |
| 137 | CHEK2 | Serine/threonine-protein kinase Chk2 |
| 138 | CLDN4 | Claudin-4 |
| 139 | PPARA | Peroxisome proliferator-activated receptor alpha |
| 140 | PPARD | Peroxisome proliferator-activated receptor delta |
| 141 | HSF1 | Heat shock factor protein 1 |
| 142 | CRP | Cysteine-rich protein 2-binding protein |
| 143 | CXCL10 | C-X-C motif chemokine 10 |
| 144 | SHUK | Inhibitor of nuclear factor kappa-B kinase subunit alpha |
| 145 | SPP1 | Sphingosine-1-phosphate phosphatase 1 |
| 146 | RUNX2 | Runt-related transcription factor 2 |
| 147 | RASSF1 | Ras association domain-containing protein 1 |
| 148 | E2F1 | Transcription factor E2F1 |
| 149 | E2F2 | Transcription factor E2F2 |
| 150 | ACP3 | Prostatic acid phosphatase |
| 151 | CTSD | Cathepsin D |
| 152 | IGFBP3 | insulin-like growth factor-binding protein 3 |
| 153 | IGF2 | Insulin-like growth factor II |
| 154 | CD40LG | CD40 ligand |
| 155 | IRF1 | Interferon regulatory factor 1 |
| 156 | ERBB3 | Receptor tyrosine-protein kinase erbB-3 |
| 157 | PON1 | Serum paraoxonase/arylesterase 1 |
| 158 | PCOLCE | Procollagen C-endopeptidase enhancer 1 |
| 159 | NPEPPS | Puromycin-sensitive aminopeptidase |
| 160 | HK2 | Hexokinase-2 |
| 161 | NKX3-1 | Homeobox protein Nkx-3.1 |
| 162 | RASA1 | Ras GTPase-activating protein 1 |
| 163 | HTR3A | 5-hydroxytryptamine receptor 3A |
| 164 | ADRA1A | Alpha-1A adrenergic receptor |
| 165 | CHRNA7 | Neuronal acetylcholine receptor subunit alpha-7 |
| 166 | IGHG1 | Immunoglobulin heavy constant gamma 1 |
| 167 | NCOA1 | Nuclear receptor coactivator 1 |
| 168 | DRD1 | D (1A) dopamine receptor |
| 169 | CHRM3 | Muscarinic acetylcholine receptor M3 |
| 170 | CHRM5 | Muscarinic acetylcholine receptor M5 |
| 171 | CHRM4 | Muscarinic acetylcholine receptor M4 |
| 172 | OPRD1 | Delta-type opioid receptor |
| 173 | OPRM1 | Mu-type opioid receptor |
| 174 | FASN | Fatty acid synthase |
| 175 | EDNRA | Endothelin-1 receptor |
| 176 | EDN1 | Endothlin-1 |
| 177 | NPM1 | Nucleophosmin |
| 178 | ECE1 | Endothelin-converting enzyme 1 |
| 179 | PARP4 | Protein mono-ADP-ribosyltransferase PARP4 |
| 180 | CALCR | Calcitonin receptor |
| 181 | ITGB3 | Intergrin beta-3 |
| 182 | CA2 | Intercellular adhesion molecule 2 |
| 183 | ADRA1D | Alpha-1D adrenergic receptor |
| 184 | STAT3 | Signal transducer and activator of transcription 3 |
| 185 | APP | Amyloid-beta precursor protein |
| 186 | ESR1 | Estrogen receptor |
| 187 | DRD2 | D (2) dopamine receptor |
| 188 | CDK2 | Cyclin-dependent kinase 2 |
| 189 | PIM1 | Serine/threonine-protein kinase pim-1 |
| 190 | NR3C1 | Glucocorticoid receptor |
| 191 | ESR2 | Estrogen receptor beta |
| 192 | GSK3B | Glycogen synthase kinase-3 beta |
| 193 | CHEK1 | Serine/threonine-protein kinase Chk1 |
| 194 | CCNA2 | Cyclin-A2 |
| 195 | PTPN1 | Tyrosine-protein phosphatase non-receptor type 1 |
